# Practical Notes on Diagnosis and Treatment

**Published:** 1909-08-28

**Authors:** 


### PRACTICAL NOTES ON DIAGNOSIS AND TREATMENT.

### Camphor in Heart Disease.

A combination of powdered digitalis leaves 1½ grains with camphor 1 grain, is useful in many cases of commencing cardiac failure. In urgent cases a grain or more of camphor dissolved in olive oil may be injected subcutaneously.

### An Incompatibility.

If calomel or yellow oxide of mercury is ordered as a local application in conjunctivitis, etc., iodine in any form should not be prescribed. When this rule is forgotten the iodine excreted by the lachrymal gland forms a very irritating compound with the mercury, and this may cause much trouble. In practice, therefore, under the above conditions, while syrup of phosphate of iron would be a suitable tonic the syrup of iodide of iron is contra-indicated.

### The Treatment of Warts.

Many cases of multiple warts successfully treated by the administration of magnesium sulphate are on record. A drachm of the sulphate should be ordered two or three times a day, and the treatment should, if necessary, be continued for some six or eight weeks.

### Alcohol in Chronic Nephritis.

Alcohol in general is to be forbidden; its transitory uses are indicated where there is lack of appetite and disturbance of the heart. A small amount of champagne in the evening may prevent the nightly attacks of uræmia and cardiac asthma, but it should be forbidden as a table beverage. It threatens the heart and the walls of the blood-vessels.—*Dr. W. B. Warrington.*

